# Diagnostic Accuracy of a 64-Slice Multi-Detector CT Scan in the Preoperative Evaluation of Periampullary Neoplasms

**DOI:** 10.3390/jcm7050091

**Published:** 2018-04-27

**Authors:** Shahryar Hashemzadeh, Behzad Mehrafsa, Farzad Kakaei, Reza Javadrashid, Rosa Golshan, Fatemeh Seifar, Farid Hajibonabi, Farzad Salmannezhad Khorami

**Affiliations:** 1 Department of General & Vascular Surgery, Imam Reza Hospital, Tabriz University of Medical Sciences, Tabriz 5166/15731, Iran; shahriar_90@yahoo.com (S.H.); fkakaei@tbzmed.ac.ir (F.K.); 2 Department of Radiology, Imam Reza Hospital, Tabriz University of Medical Sciences, Tabriz 5166/15731, Iran; rjrashid@hotmail.com (R.J.); rosa3606@yahoo.com (R.G.); 3 Student Research Committee, Tabriz University of Medical Sciences, Tabriz 5166/15731, Iran; f.seifar@gmail.com (F.S.); f.bonabi@yahoo.com (F.H.); 4 Department of Emergency Medicine, Imam Reza Hospital, Tabriz University of Medical Sciences, Tabriz 5166/15731, Iran; medicsonf@gmail.com

**Keywords:** multi-detector computed tomography, diagnosis, periampullary carcinoma, pancreas

## Abstract

Periampullary tumors are highly malignant masses with poor prognosis. Surgical resection is the only treatment for patients with this disease. The preoperative evaluation of masses is essential to determine the tumor resectability and vascular invasion. The aim of this study was to determine the diagnostic accuracy of 64-slice multi-detector computed tomography (MDCT) in detecting the resectability of periampullary masses. A cross-sectional study was conducted on patients with a definite diagnosis of periampullary cancer. All the participants underwent an MDCT scan before the surgical pancreaticoduodenectomy. The preoperative results were compared to the intraoperative findings and the diagnostic accuracy was determined based on the sensitivity and specificity of the MDCT. From June 2015 until June 2016, 32 patients with periampullary carcinoma were enrolled in the study. Of 32 masses, one of them considered nonresectable because of the gross vascular invasion in th CT images. After the operation, the overall resectability rate was 81.3%. The sensitivity and specificity of MDCT for tumor resectability was 100% and 16.7%, respectively, with an overall accuracy of 84.4%. To sum up, MDCT had high sensitivity but low specificity in the preoperative evaluation of preampullary carcinomas. The low specificity resulted from the low accuracy of the CT scan in detecting vascular involvement.

## 1. Introduction

Pancreatic cancers are the fourth leading cause of cancer-related mortality in the world. The overall survival rate is poor; the five-year survival rate is less than five percent [1,2]. Periampullary neoplasms refer to masses located near the ampulla of Vater, which originate from the pancreas, ampulla, duodenum or common bile duct [3]. Periampullary neoplasms are known as the deadliest cancers, for which surgical resection is the most effective curative treatment. The poor prognosis for pancreatic cancer is due to few or non-specific symptoms in the early stages of the disease. The precise screening and preoperative staging rely on the imaging modalities [4,5].

However, the imaging techniques cannot always detect masses of the periampullar area in primary stages or make a definitive diagnosis.

Currently, the great advances in operating methods have helped to improve the survival rate. Pancreaticoduodenectomy is the treatment of choice for surgical approaches. The most essential surgical step is the initial evaluation of tumors to differentiate the resectable masses from those with progressive vascular encasement [6,7].

In clinical practice, computed tomography (CT) scan is commonly used as the first preoperative examination tool in patients with suspected pancreatic cancer. It provides wide anatomic coverage with acceptable resolution as well as the evaluation of vascular involvement through a single session [8]. CT imaging plays a key role in the characterization of periampullar lesions, preoperative staging, post-surgical and therapeutic planning [9].

There are some reports in which the preoperative diagnostic accuracy of CT has been assessed. The positive predictive value of CT scans in detecting the resectability of pancreatic cancer was 89% and the sensitivity and specificity were 100% and 72%, respectively, in these studies [10,11].

Although a few studies have evaluated the predictive value of CT scans for pancreatic cancers, its preoperative accuracy for preampullary masses still remains unknown.

The aim of this study was to measure the diagnostic accuracy of CT scan in detecting the resectability of periampullary masses.

## 2. Experimental Section

This cross-sectional prospective study was conducted for a year from June 2015 until the end of June 2016 at the Imam Reza University Hospital of Tabriz, Iran. The study protocol was approved by the ethical committee of the Tabriz University of Medical Sciences.

The patients were those with presumed periampullary tumors, who were candidates for surgical resection based on the diagnosis of a specialist who had referred them to the radiology department of Imam Reza hospital. Patients were notified of the detailed instructions and aim of the project and written consent was obtained from all the participants before the study.

All the participants received a water-soluble oral contrast before the imaging process. An intravenous administration of 100 mL of non-nonionic iodinated contrast solution was performed on the patients. CT scanning was started after completion of the contrast injection. The 64-slice multi-detector abdominal CT (MDCT) was performed on the patients by means of a contrast-enhanced protocol. Enhanced views of the peripan creatic area were obtained and interpreted by two radiologists as resectable or non-resectable periampullary masses.

Preoperative images of periampullary masses obtained by CT scan were interpreted as resectable if there was a clear tissue plane between the tumor and superior mesenteric artery (SMA), non-diagnostic evidence of metastasis, and <180° circumferential involvement of the superior mesenteric vein–portal vein (SMV-PV) confluence.

The CT criteria for non-resectable tumors were abdominal ascites, SMA involvement, occlusion of the junction between the superior mesenteric vein and hepatic PV, liver metastasis, spread of tumor to the extra-pancreatic organs (excluding duodenum and colon) and peritoneum, or involvement of distant lymph nodes except for peripancreatic nodes.

The vascular involvement in the CT scan was detected based on an abrupt change to a vessel caliber with or without collateral vessels, a loss of the interface between a vessel and a tumor, and/or the presence of a tumor within a vessel.

Patients determined to have preoperatively resectable tumors underwent surgical pancreaticoduodenectomy. During the surgical procedure, tumors were evaluated by an experienced surgeon. The vascular involvement of each tumor was also determined by the surgeon based on the gross intraoperative findings and recorded. The diagnostic accuracy of the resectibablity of masses and the vascular involvement of the tumor was considered as consistency between the CT reports and intraoperative findings. The sensitivity and specificity of CT scans as well as their negative and positive predictive values were analyzed using Dag_Stat software (http://www.dagstat.de). Fair consistency between the findings was assessed by kappa. A *p* value of 0.05 and lower was considered statistically significant.

## 3. Results

In the 12 months of the study, one hundred and twelve patients suspected of pancreatic cancer were referred to the center. The patients were evaluated and thirty-two patients with a definite diagnosis of periampullary carcinoma were enrolled in the study.

**Figure 1:**
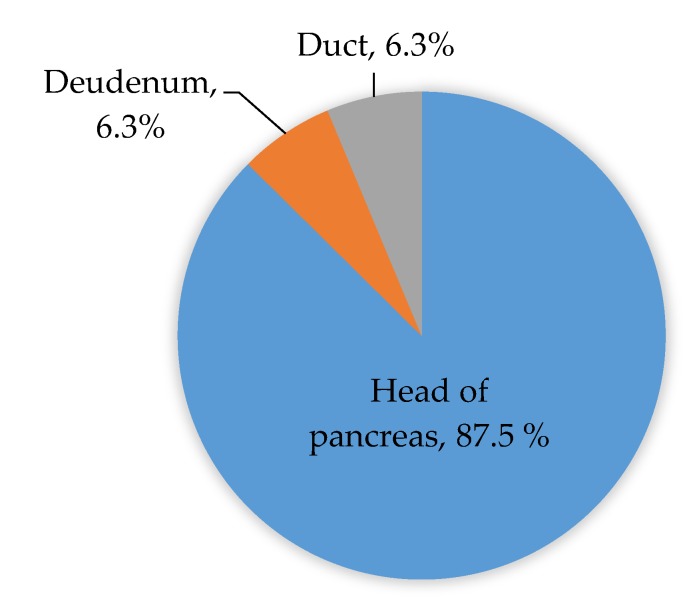
The location of the masses in the periampullary region.

Of these, 18 patients (56.3%) were male and 14 (43.8%) were female, with a mean age of 54 ± 12.5 years (range 25 to 72 years). The most common primary symptom was abdominal pain and discomfort among the patients. Table 1 shows the prevalence of the combination of non-specific symptoms among the study population. Twenty-eight patients had adenocarcinoma of the head of pancreas, two had a duodenal mass, and two had choledochal malignancy (Figure 1).

The location of the masses was determined in the periampullary area by CT scan. All the tumors were considered potentially resectable based on the CT scan findings except for one mass with gross vascular invasion. The CT scan results did not reveal metastasized organs or peritoneal and liver involvement. Vascular invasion was reported in one of the patients (3.1%), in whom the confluence of the mesenteric, celiac and portal vessels was affected by tumor implants.

Thirty-one of the participants underwent surgical operation to perform a total tumor resection. The classic pancreaticoduodenectomy was performed on the periampullary masses without vein resection.

Pancreaticoduodenectomy was performed in 26 patients, resulting in an overall resectability rate of 81.3%. The surgical findings approved the location of tumors and did not report any region of metastasis or ascites. Tumor invasion to a nearby organ was noted in one of the patients. Five more patients were found to have vascular involvement that was undetected by CT scan. Patients who were unresectable because of SMV/PV or SMA invasion were those that the surgeon thought would have positive margins even with major vascular resection. These patients included 18.8% of the participants.

Of 32 patients with highly suspicious of periampullary cancers, 31 had definitive, CT criteria for resectability, and therefore underwent an exploratory laparotomy (96.87%) (Table 2).

Of the 31 patients who underwent exploratory surgery, five (16.12%) were found to have unresectable disease due to gross vascular involvement. Table 3 indicates the predictive values, sensitivity and specificity of the 64-slice multi-detector CT for the resectability of periampullary malignancies.

The analysis using the kappa coefficient demonstrated fair correlation co-efficiency between the CT scan reports and the intraoperative results for tumor resectability (*p* = 0.03, kappa = 0.25).

In this study, tumor invasion of the SMV, SMA, hepatic PV and celiac vessel was determined during the operation and compared to the preoperative CT scan reports.

The predictive values, sensitivity and specificity of the CT scan in detecting the malignant involvement of the great splenic vessels are listed in Table 4.

## 4. Discussion

Periampullary tumors include a group of malignancies originating from the pancreas, duodenum, distal common bile duct, and ampulla of Vater. Despite the difference in origin, they all have a highly malignant nature and present in late stages, which leads to poor curative prognosis. Total surgical resection is known as the only therapeutic possibility for patients with this disease [12,13].

Unfortunately, most patients present with an advanced stage of disease, with aggressive tumors invading the confluence of major vascular structures, such as mesenteric and celiac vessels. They may also involve the lymph and nervous system and metastasize to local and distal organs. Therefore, early diagnosis and the initial evaluation of tumor invasion plays an essential part in the treatment approach [14,15]. The imaging modalities provide a preoperative evaluation to determine the tumor extension to nearby organs as well as its spread to regional lymph nodes, major vessels, and distant organs. This information would help in the determination of the resectabality of tumors during surgical procedures [16,17]. In this single-center prospective study, we tried to measure the predictive values of 64-slice MDCT images in the preoperative evaluation of periampullary masses.

Although our findings are representative of a limited patient population, we showed that MDCT has a high sensitivity and accuracy rate in identifying the resectability of periampullary malignancies.

In a group of 32 patients with periampullary cancer, possible resectability was reported for 31 of them. The rate of surgical resection was 81.25% in this study. The false positive results represented 15.6% of participants. The intraoperative discovery found five unresectable patients that were not detected by MDCT scan images. These results led to specificity as low as 16.7% for the CT scan.

Tomazic et al. have also indicated a similar low specificity for CT scans in such tumors, namely 45.8% [18]. On the other hand, Howard et al., found a sensitivity of 63%, specificity of 100% and an overall accuracy of 86% for CT scans of 21 patients with periampullary carcinomas [19].

The sensitivity of MDCT for predicting the resectability of periampillary masses in the present study was higher than the publications in the literature. Other studies have reported a range of 33% to 63% for CT scan sensitivity [19,20,21].

The predictive negative and positive values were 100 and 83.9% for the MDCT scans in our study. These results were superior to other studies that reported a predictive value of 70% to 80% for spiral CTs.

Lee et al. reviewed the accuracy of different imaging methods for pancreatic malignancies, indicating that distinct advances in CT technology, especially the use of MDCT for detection, diagnosis and staging have caused great improvements in the preoperative evaluation of pancreatic cancers [10].

Furthermore, some studies have compared the diagnostic value of MDCT with magnetic resonance imaging (MRI), which was found to be similar for the detection of malignancies in the pancreatic region.

Koelblinger et al. reported 95% sensitivity and 96% specificity for 64-slice MDCT, as well as 96% sensitivity and 96% specificity for 3.0-T MRI in diagnosing pancreatic masses [22].

In this study, MDCT images and diagnostic surgery revealed identical results in detecting metastasis and peritoneal and lymph node involvement, while based on other results, MDCT may not depict small metastases to the peritoneum or liver, or even a primary pancreatic mass [23,24].

Since the 1990s, the CT criteria of vascular involvement have been described for the preoperative evaluation of periampullary masses. These criteria were improved by the emerging feature of MDCTs in clinical studies [25,26].

In the present study, MDCT images could detect only one of the six gross vascular invasions found by discovery operation. It led to a sensitivity of 16.7% for CT scans in our study, while a high specificity of 100% was determined for detecting vascular involvement.

In 2004, Vargas et al. determined the accuracy of MDCT in the evaluation of vascular involvement in pancreatic and periampullary tumors. They performed a retrospective study on 22 patients who underwent pancreaticoduodenectomy and found a negative predictive value of 100% with no false negative results, and an accuracy of 99% for MDCT in detecting vascular invasion. The negative predictive value was 83.9% in our study [15]. In another study by House et al., the diagnostic accuracy of 3D CT scans was assessed for the vascular involvement of different vessels. They found the accuracy of 90% for the superior mesenteric vein and portal vein and an accuracy of 95% and 98% for the superior mesenteric artery and celiac trunk, respectively [27]. Similarly, Manak et al. reported a negative predictive value of 99% for biphasic MDCT for the detection of vessel involvement of pancreatic cancer [28]. We also found an overall accuracy of 84.4% for vascular involvement in this study. This discrepancy in the literature compared to our findings may be due to the different sample of the population, the methodology of using a retrospective approach, different types of the CT scans and the molecular tracer.

In this study, the 64-slice MDCT had a high sensitivity but low specificity in the preoperative evaluation of preampullary carcinomas. The low specificity resulted from the low accuracy of CT scans in detecting vascular involvement.

Therefore, MDCT is still the modality of choice for the diagnosis and pre-operative evaluation of patients with periampullary cancers, while the combination of CT scans with other imaging modalities, especially CT angiography, is suggested for better detection of vascular involvement.

## Figures and Tables

**Table 1:** Frequency of the patients’ symptoms among the study population.

Symptoms	Frequency	Percent
Abdominal pain, nausea, vomiting, anorexia, icterus	20	62.5
Abdominal pain, nausea, vomiting, icterus	8	25
Abdominal pain, nausea, anorexia, icterus	2	6.3
Abdominal pain, anorexia, icterus	2	6.3

**Table 2:** The correlation between the preoperative CT scan findings and exploratory operation.

		CT Scan		
		Resectable	Non-resectable	Total
**Operation**	Resectable	26	0	26
	Non-resectable	5	1	6
	Total	31	1	32

**Table 3:** The diagnostic value of the preoperative CT scan in the reseactability of tumors.

Predictor	Percent
Sensitivity	100
Specificity	16.7
PPV	83.9
NPV	100
Diagnostic accuracy	84.4

**Table 4:** The diagnostic value of preoperative CT scan in the vascular invasion of the tumor.

Predictor	Percent
Sensitivity	16.7
Specificity	100
PPV	100
NPV	83.9
Diagnostic accuracy	84.4
